# Lymphoid Organs Remodeling in Non‐Obese Diabetic Goto‐Kakizaki Rats Immunized With SARS‐CoV‐2 Antigens

**DOI:** 10.1096/fj.202503631R

**Published:** 2026-01-24

**Authors:** Sara Araujo Pereira, Mariana Cruz Lazzarin, Maria Lucia Zaidan Dagli, Bianca de Carvalho Lins Fernandes Távora, Eliana Faquim de Lima Mauro, Marcelo Larami Santoro, Sandra Coccuzzo Sampaio Vessoni, Luis Roberto de Camargo Gonçalves, Marina Sakamoto Sotoyama, Ricardo das Neves Oliveira, Vania Gomes de Moura Mattaraia, Rui Curi

**Affiliations:** ^1^ Laboratory of Pathophysiology Butantan Institute São Paulo Brazil; ^2^ Interdisciplinary Post‐Graduate Program in Health Sciences Cruzeiro do Sul University São Paulo Brazil; ^3^ University of Ribeirão Preto Guarujá Brazil; ^4^ Department of Pathology, School of Veterinary Medicine and Animal Science University of São Paulo São Paulo Brazil; ^5^ Laboratory of Immunopathology Butantan Institute São Paulo Brazil; ^6^ Bioterio Central Butantan Institute São Paulo Brazil; ^7^ Bioindustrial Center Butantan Institute São Paulo Brazil; ^8^ Butantan Institute School Butantan Institute São Paulo Brazil; ^9^ University of Marilia Marilia Brazil

**Keywords:** antibodies, B‐lymphocytes, humoral, immunity, metabolic diseases, morphometry, vaccination

## Abstract

The COVID‐19 pandemic revealed reduced vaccine efficacy and worse outcomes in people with type 2 diabetes mellitus (T2DM). However, how T2DM, independent of obesity, alters immune responses and lymphoid organ architecture remains unclear. We evaluated antibody production and lymphoid organ morphology following SARS‐CoV‐2 antigen immunization in Goto‐Kakizaki (GK) rats, a non‐obese T2DM model, to evaluate the isolated effect of diabetes. Ten‐week‐old male Wistar and GK rats received three intramuscular injections of SARS‐CoV‐2 antigens at 15‐day intervals. Two antigen formulations were used: chemically inactivated whole SARS‐CoV‐2 containing aluminum hydroxide adjuvant and an inactivated Newcastle virus engineered to express the SARS‐CoV‐2 spike protein. Fifteen days after the final dose, anti‐Spike IgG titers were measured by ELISA, and histological and morphometric analyses of lymphoid tissues were performed. GK rats exhibited elevated anti‐Spike IgG responses compared with WT controls, regardless of the antigen formulation. Distinct morphological alterations in the lymphoid organs accompanied the enhanced IgG responses. Mesenteric lymph nodes from GK rats exhibited a morphology similar to that of WT rats. The thymus exhibited features of involution, including septal thickening, reduced corticomedullary distinction, diminished cortical cellularity, cortical thinning, and medullary expansion. The spleen showed white pulp remodeling, characterized by enlargement of germinal centers and periarteriolar lymphoid sheaths, along with a reduction in the marginal zone. We described herein the remodeling of the primary and secondary lymphoid organs in non‐obese diabetic GK rats, which may play a role in the antibody response to an immune challenge.

## Graphical abstract

The Goto‐Kakizaki rat, a non‐obese model of type 2 diabetes mellitus (T2DM), exhibits lymphoid organ remodeling, including baseline thymic involution and splenic white pulp reorganization. Following completion of a three‐dose SARS CoV‐2 antigen immunization protocol, anti‐Spike IgG levels were significantly increased despite these structural changes. These findings show that, in the period analyzed, chronic hyperglycemia reshapes lymphoid architecture without impairing humoral immunity, supporting precision vaccinology and personalized immune interventions in diabetes. Created in https://BioRender.com.

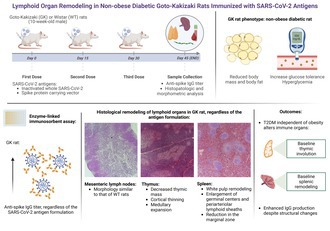

## 1 Introduction

Since the emergence of the COVID‐19 pandemic in 2019, it has become evident that individuals with pre‐existing comorbidities, such as obesity and type 2 diabetes mellitus (T2DM), exhibit a worse prognosis following SARS‐CoV‐2 infection [1, 2]. Indeed, people with diabetes, especially those with uncontrolled glycemic levels, have a higher risk of hospitalization, intensive care unit admission, and mortality [3, 4].

During the pandemic, pre‐existing T2DM was associated with a two‐fold increase in the risk of severe or critical COVID‐19 and a threefold increase in in‐hospital mortality [5]. These vulnerabilities are largely attributed to chronic low‐grade inflammation, enhanced viral replication during hyperglycemia, and the increased angiotensin‐converting enzyme 2 (ACE2) receptor expression. Elevated glucose levels can glycate both ACE2 and the viral spike (S) protein, potentially increasing binding affinity and facilitating viral entry. Following S protein binding and protease‐mediated priming (TMPRSS2, cathepsins), ACE2 downregulation disrupts the renin–angiotensin–aldosterone system, promoting vasoconstriction, oxidative stress, and inflammation [6]. Direct endothelial infection further amplifies vascular injury, contributing to worse outcomes for individuals with T2DM [1, 7].

Several vaccines have been developed, including inactivated virus vaccines, viral vector vaccines, and mRNA‐based platforms, and distributed globally to mitigate the impact of the COVID‐19 pandemic [8]. Although these vaccines have proven effective in the general population [9], their immunogenicity in individuals with T2DM remains controversial.

For example, studies have reported reduced antibody (IgG) titers, lower neutralizing capacity, and decreased seroconversion rates in individuals with diabetes, regardless of the vaccine or vaccine protocol used. This diminished response may be further attenuated in obese diabetic individuals due to chronic low‐grade inflammation associated with excessive adiposity [10]. Additionally, low vaccine efficacy in T2DM has been linked to dysfunctional T‐cell activity and reduced B‐cell activation, both of which are crucial for effective antibody production [11].

The Goto‐Kakizaki (GK) rat is a well‐established, non‐obese model of spontaneous T2DM [12], characterized by persistent hyperglycemia and peripheral insulin resistance, followed by progressive alterations in pancreatic β‐cell mass [13, 14]. Following its original development in Japan, GK rat colonies were established worldwide, and sub‐strains with different pancreatic islet features have been identified [15].

Immune alterations have been reported in GK rats; however, these findings may vary depending on the biological compartment analyzed, the immunological endpoints assessed, the age of the animals, and colony‐specific features. Seal et al. [16] have reported reduced circulating immune cell numbers and low systemic cytokine levels in six‐month‐old GK rats [16]. Silveira et al. [17] and Lobato et al. [18] described tissue‐ and cell‐specific immune changes in younger GK rats, highlighting differences in immune cell composition, activation, and polarization. The same reported above for pancreatic islets may also apply to the immune system and so requires investigation.

It is noticeable that the structural and histological features of their lymphoid organs, particularly the thymus, spleen, and mesenteric lymph nodes, remain poorly investigated in GK rats. These organs play a central role in the development and regulation of adaptive immunity, serving as primary sites for antigen presentation and lymphocyte activation [19, 20, 21]. Morphological alterations in these organs may disrupt the activation and differentiation of immune cells, impairing the generation of effective vaccine‐induced responses. Thus, investigating how diabetes alters lymphoid tissue structure in GK rats is crucial to understanding the mechanisms that explain altered immunogenicity in diabetic individuals.

In the present study, we evaluated histomorphometry alterations in the thymus, spleen, and mesenteric lymph nodes of GK rats following immunization with SARS‐CoV‐2 antigens. By comparing diabetic GK rats to normoglycemic Wistar controls, we sought to isolate the effects of diabetes per se, without considering the impact of obesity.

## 2 Material and Methods

### 2.1 Animals and Experimental Groups

Ten‐week‐old male *Rattus norvegicus* from the Wistar (WT) and Goto–Kakizaki (GK) strains, weaned at 21 days of age, were distributed into six groups: WT + Saline (WT‐S); WT + Inactivated whole SARS‐CoV‐2 (WT‐IWS); WT + Spike protein carrying vector (WT‐SPV); GK + Saline (GK‐S); GK + Inactivated whole SARS‐CoV‐2 (GK‐IWS); GK + Spike protein carrying vector (GK‐SPV).

The animals used in this study were obtained from the Animal Welfare Center of the Butantan Institute (São Paulo, Brazil) and subsequently housed in the animal maintenance room of the Laboratory of Physiopathology of the Butantan Institute. The GK breeding stock maintained at the Animal Welfare Center of the Butantan Institute was originally imported from Taconic Bioscience (Rensselaer, NY, USA) to establish our colony.

The animals were maintained under controlled conditions, with a temperature range of 22°C–24°C, a 12‐h light–dark cycle, and free access to water and standard rodent chow (Nuvilab, Curitiba, Paraná, Brazil).

All experiments were approved by the Ethics Committees at the Butantan Institute (CEUAIB; No. 3071280322) and Cruzeiro do Sul University (No. 005/2024).

### 2.2 Glucose Tolerance Test

A glucose tolerance test (GTT) was performed 5 days before starting immunization. After a 6‐h fast, animals were administered a 50% glucose solution intraperitoneally at a dose of 2 g/kg body weight. Blood samples were obtained from a tail tip cut before (0 min) and 15, 30, 60, and 90 min after glucose injection [22]. Blood glucose concentration was determined using an Accu‐Chek blood glucose monitor (Roche, São Paulo, Brazil).

### 2.3 Immunization

Two antigen formulations were used for immunization: a chemically inactivated whole SARS‐CoV‐2 virus adjuvanted with aluminum hydroxide (IWS) [23] and an inactivated Newcastle disease virus engineered to express the SARS‐CoV‐2 S protein (SPV) [24]. Animals were immunized intramuscularly with three doses of 10 μL of IWS antigen or 5 μL of SPV antigen, administered at 15‐day intervals. The dosage (mg/kg) was calculated to be equivalent to that used in humans during the COVID‐19 pandemic. As a reference, the dose corresponded to 3 μg of antigen in 0.5 mL of the vehicle used in the vaccine preparation [25, 26]. The equivalent human dose was determined based on body mass and metabolic rate, following the method by Nair and Jacob [27].

At 2 weeks post‐immunization, rats were euthanized, and blood and lymphoid organs were collected.

### 2.4 Enzyme‐Linked Immunosorbent Assay

S protein‐specific IgG antibody titers in rat samples were measured by ELISA. Briefly, 96‐well flat‐bottomed plates (Nunc, Roskilde, Denmark) were coated with 100 μL of vaccine preparation containing 2 μg of antigen per well and incubated overnight at 4°C. Plates were washed with 0.05% Tween 20 Phosphate Buffered Saline (PBS) using a plate washer (Stat Fax 2600, Awareness Technology Inc., Palm City, FL, USA). Subsequently, 200 μL of blocking solution (3% skim milk diluted in PBS‐Tween) was added to each well, and the plates were incubated at 37°C for 2 h. After blocking, three additional washes with PBS‐T were performed. Serum samples were serially diluted from 1:200 to 1:25 600 and then incubated for 2 h at 37°C. Plates were washed three times and incubated for 2 h at 37°C with peroxidase‐conjugated anti‐rat IgG secondary antibody (A‐9037 Sigma) diluted 1:5000 in PBS‐T. Plates were washed three times and developed with solution [33 mM citrate–phosphate buffer, pH 5; O‐phenylenediamine dihydrochloride (OPD); and hydrogen peroxide (H_2_O_2_)] for 15 min at room temperature, in the dark. The reaction was stopped with 1 M sulfuric acid. The plates were read at 492 nm using a plate reader. Antibody levels were expressed as the area under the curve (AUC) [28].

### 2.5 Collection of the Lymphoid Organs

Lymphoid organs were weighed immediately after collection. The results were expressed as both absolute organ weight (g) and as relative organ weight normalized to body mass (g/100 g body weight).

The thymus, spleen, and mesenteric lymph nodes were collected and immediately fixed for 24 h in 10% phosphate‐buffered formaldehyde. After fixation, the specimens were dehydrated in an ascending ethanol series and embedded in paraffin. Semi‐serial 4 μm sections were obtained using an RM2155 microtome (Leica Microsystems, Wetzlar, Germany). Each histological slide contained three sections and was stained with hematoxylin and eosin (H&E) for both histopathological and morphometric evaluations.

### 2.6 Histopathological and Morphometric Analysis

Photomicrographs for histopathological and morphometric analyses were acquired using a Leica DMLB microscope equipped with a Leica DFC420 digital camera and LAS v4.5 imaging software (Leica Microsystems).

Histopathological analysis was performed on two sections per animal, following the criteria described by Willard–Mack et al. [29]. This analysis included the location and organization of tissue compartments, cellularity in each region, cell density, and morphological features such as cellular hypertrophy and atrophy. Representative histological plates were assembled to illustrate the predominant qualitative features observed in each group.

The semi‐quantitative analysis of lymphoid organ structures was performed according to the stereological principles established by Mandarim‐De‐Lacerda [30], using specific protocols tailored to each organ. The volume density (Vv) of the selected tissue compartments was estimated using the point‐counting method, with test grids superimposed on photomicrographs analyzed in ImageJ software (National Institutes of Health, Bethesda, MD, USA). For each image, the total number of test points (Pt) intersecting the reference area and the number of points (Pp) falling on the target structure were recorded. The volume density was calculated using the formula: Vv = ƩPp/ƩPt.

The expression of tissue compartments as relative volume densities accounts for differences in organ size across animals, enabling direct comparison of lymphoid organ architecture [30].

- Thymus: Five photomicrographs per animal were captured using a 4× objective lens, covering the entire extent of the tissue section. A test grid with 216 intersections was applied to estimate the volume density of the cortex and medulla.
- Spleen: Five photomicrographs per animal were captured using a 4× objective lens across the full length of the splenic section. Volume density of the red pulp and white pulp was estimated using a 216‐point test grid. For a detailed assessment of white pulp compartments, marginal zone, lymphoid follicle, germinal center, and periarteriolar lymphoid sheath (PALS), five additional images per animal were captured using a 10× objective lens and a test grid with 240 points.
- Mesenteric lymph node: Five photomicrographs per animal were captured using a 4× objective lens to analyze cortex, paracortex, and medulla regions with a 216‐point grid. For follicular analysis, 20 lymphoid follicles per animal were selected and imaged using a 10× objective lens. To account for differences in lymph node size between animals, follicle and germinal center areas were normalized to the total mesenteric lymph node mass of each animal and expressed as square millimeters (mm^2^) of histological area per gram of lymph node tissue.

### 2.7 Statistical Analysis

Statistical analyses were performed using GraphPad Prism v8.0 (GraphPad Software, San Diego, CA, USA). Data normality was assessed using the Shapiro–Wilk test. Comparisons among groups were conducted using two‐way analysis of variance (ANOVA), followed by Tukey's multiple comparisons post hoc test. Results were expressed as mean ± standard error of the mean (SEM), and differences were considered statistically significant at *p* ≤ 0.05.

## 3 Results

### 3.1 GK Rats Exhibit Decreased Body Mass, Length, and Accumulation of Fat Mass, but Increased Hyperglycemia

**FIGURE 1:**
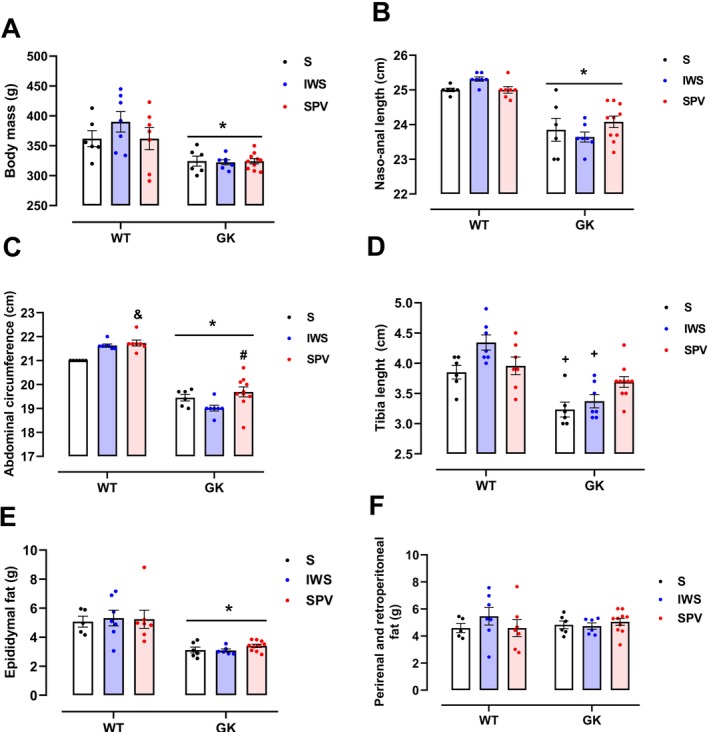
Body composition and morphometric parameters of Wistar (WT) and Goto‐Kakizaki (GK) rats following immunization with saline (S), inactivated whole SARS‐CoV‐2 virus (IWS), or spike protein carrying vector (SPV). All measurements were obtained from 16‐week‐old rats, 15 days after completion of the immunization protocol. (A) Body mass; (B) Naso–anal length; (C) Abdominal circumference; (D) Tibia length; (E) Epididymal fat mass; (F) Perirenal and retroperitoneal fat mass. Results are presented as mean ± SEM (*) *p* < 0.05: between strains; (#) *p* < 0.05: between immunized animals (same strain); (&) *p* < 0.05: versus saline group (same strain); (+) *p* < 0.05: same treatment, different strains.

After the 45‐day experimental protocol, GK rats exhibited a 12.8% lower body mass (Figure 1A) and a 4.94% shorter naso–anal length (Figure 1B) than WT rats (*p* < 0.0001 for both comparisons), regardless of the antigen administered. Similarly, the GK group exhibited a 9.65% lower average abdominal circumference than the WT group (*p* < 0.0001), irrespective of the antigen (Figure 1C). In the WT group, animals immunized with SPV had a significantly greater abdominal circumference than those receiving saline (*p* = 0.0270). In the GK group, a significant difference was observed between the IWS‐ and SPV‐immunized groups, with lower values in the SPV group (*p* = 0.0183). Regarding tibia length (Figure 1D), saline‐treated GK rats exhibited significantly shorter tibias compared with WT rats under the same conditions (*p* = 0.0177). This difference was even more pronounced in the IWS‐immunized groups, with GK rats showing significantly lower values (*p* < 0.0001). In terms of adiposity, the GK group displayed 38.5% less epididymal fat deposition (Figure 1E) compared with the WT group, regardless of the antigen administered. No statistically significant differences in perirenal and retroperitoneal fat were detected between groups (Figure 1F).

**FIGURE 2:**
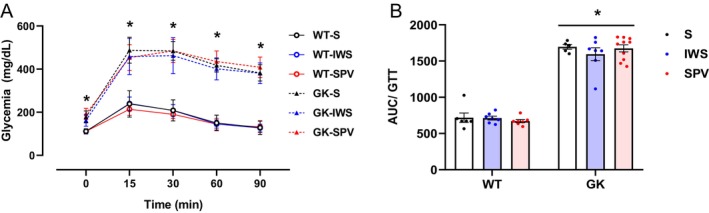
Glycemic curve during glucose tolerance test (GTT), and area under the curve (AUC) of glycemia in Wistar (WT) and Goto‐Kakizaki (GK) at baseline, before the immunization protocol. All analyses were performed in 10‐week‐old rats. (A) Glycemia was measured at baseline and 15, 30, 60, and 90 min after glucose administration. (B) AUC analyses of the GTT results for WT and GK rats. Results are presented as mean ± SEM (*) *p* < 0.05: between strains at the same time point.

Before the immunization protocol, animals were challenged with glucose (2 g/kg body weight) to assess glucose tolerance and confirm the hyperglycemic profile in the different experimental groups. GK rats exhibited significantly higher blood glucose levels at all time points compared with WT rats (*p* < 0.0001), regardless of the administered antigen (Figure 2A). At baseline (0 min), GK rats already presented elevated glycemia, which further increased after glucose administration, reaching a peak between 15 and 30 min, with values exceeding 500 mg/dL. In contrast, WT rats showed a moderate increase in glycemia, with a peak below 300 mg/dL, followed by a gradual return to baseline within 90 min. No statistically significant differences were observed between subgroups. The AUC analysis of the glucose tolerance test confirmed a significantly impaired glucose metabolism in GK rats compared with WT rats (*p* < 0.0001) (Figure 2B).

### 3.2 Treatment Elicits IgG Production and Anti‐Spike IgG Titers Are Higher in GK Rats, Independent of the SARS‐CoV‐2 Antigen Formulation

**FIGURE 3:**
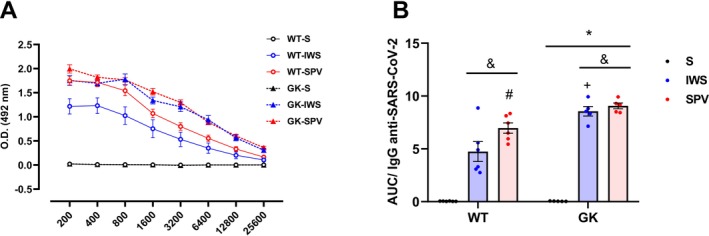
Serum anti‐spike IgG responses in Wistar (WT) and Goto‐Kakizaki (GK) rats following immunization with saline (S), inactivated whole SARS‐CoV‐2 virus (IWS), or spike protein–carrying vector (SPV). All analyses were performed in 16‐week‐old rats, with serum samples collected 15 days after the final immunization dose. (A) Anti‐spike IgG levels were evaluated in serum by ELISA using serial dilutions. (B) Area under the curve (AUC) quantification of anti‐spike IgG responses. Results are presented as mean ± SEM. (*) *p* < 0.05: between strains; (#) *p* < 0.05: between immunized animals (same strain); (&) *p* < 0.05: versus saline group (same strain); (+) *p* < 0.05: same treatment, different strains.

Anti‐spike protein antibody levels were measured 15 days after the third and final dose of antigen, completing the immunization protocol. This assessment aimed to determine whether the treatment effectively induced IgG antibody production by administering SARS‐CoV‐2 antigens. In the serial dilution assay (Figure 3A), optical density (OD) values increased in the antigen‐immunized groups compared with the saline controls, which exhibited minimal OD readings, indicating an absence of anti‐spike IgG production. In the immunized animals, OD values decreased progressively with increasing serum dilution, consistent with a specific, measurable antibody response.

The AUC analysis for anti‐spike IgG levels (Figure 3B) confirmed that both antigens elicited robust IgG responses in both strains when compared with saline‐treated animals (*p* < 0.0001), with GK rats exhibiting significantly higher titers than WT rats (*p* < 0.0001), regardless of the antigen administered. In WT rats, the S protein‐carrying vector induced IgG levels 46.8% higher than those elicited by the inactivated SARS‐CoV‐2 formulation (*p* = 0.0002).

### 3.3 Morphology of Mesenteric Lymph Nodes (MLNs) From GK Is Similar to That of WT Rats

**FIGURE 4:**
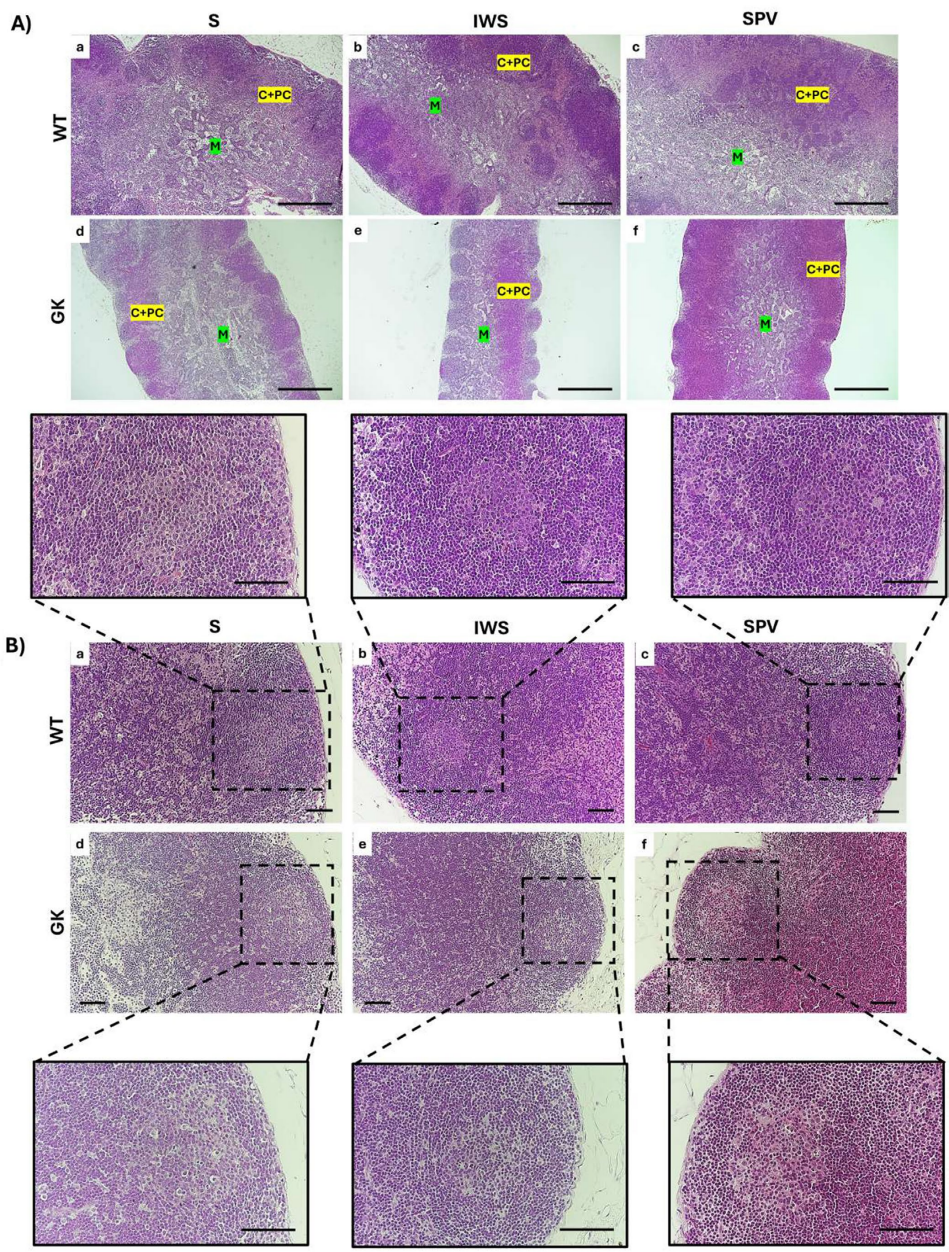
Histopathological evaluation of the mesenteric lymph nodes (MLNs) in Wistar (WT) and Goto‐Kakizaki (GK) rats following immunization with saline (S), inactivated whole SARS‐CoV‐2 virus (IWS), or spike protein–carrying vector (SPV). All analyses were performed in 16‐week‐old rats, with MLNs collected 15 days after completion of the immunization protocol. (A) Architecture of the MLNs, with demarcated cortical + paracortical (C + PC) and medullary (M) regions. (B) Representative images of lymphoid follicles in the cortical region. High‐magnification details secondary lymphoid follicles with germinal centers of WT and GK animals. Scale bars: (A) 500 μm; (B) 100 μm; (high‐magnification photomicrographs) 50 μm.

At baseline, GK exhibited MLNs with histological organization, characterized by distinct cortical, paracortical, and medullary regions, similar to those of WT rats (Figure 4A). This organization was not altered by immunization. The cortex, located in the subcapsular region, contained both primary lymphoid follicles, lacking germinal centers, and secondary lymphoid follicles with well‐defined germinal centers. The medulla, adjacent to the hilum, lacked follicles, and the paracortex was situated between these two regions.

At baseline, GK rats exhibited a slightly lower proportion of secondary lymphoid follicles (62.75%) compared with WT rats (73.04%), and this distribution was not altered following immunization (Figure 4B).

**FIGURE 5:**
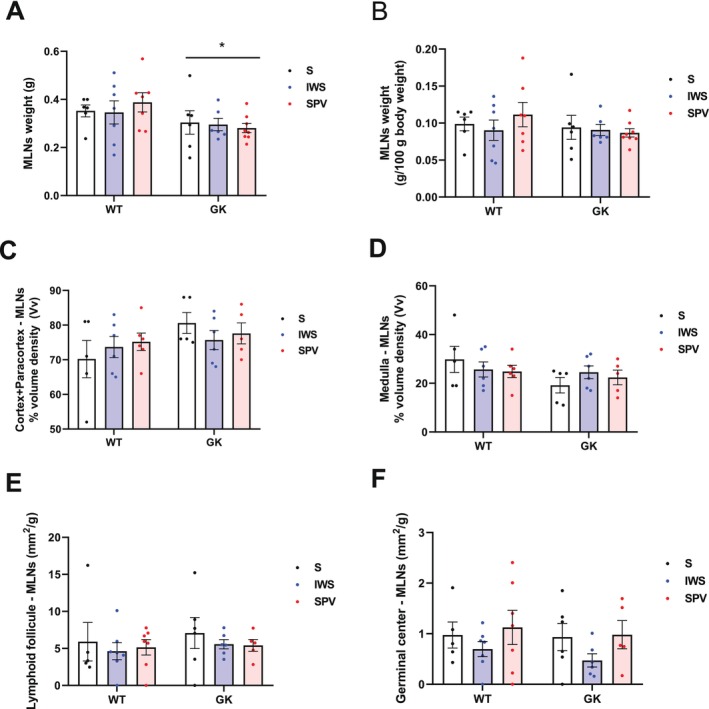
Morphometric analysis of the mesenteric lymph nodes (MLNs) in Wistar (WT) and Goto‐Kakizaki (GK) rats following immunization with saline (S), inactivated whole SARS‐CoV‐2 virus (IWS), or spike protein carrying vector (SPV). All analyses were performed in 16‐week‐old rats, with MLNs collected 15 days after completion of the immunization protocol. (A) Absolute MLN weight (g). (B) MLN weight normalized to body mass (g/100 g body weight). (C) Volume density (%Vv) of the cortical and paracortical regions. (D) Volume density (%Vv) of the medullary region. (E) Lymphoid follicle area normalized to MLN mass (mm^2^/g). (F) Germinal center area normalized to MLN mass (mm^2^/g). Results are presented as mean ± SEM. (*) *p* < 0.05: between strains. Results are presented as mean ± SEM (*) *p* < 0.05: between strains.

Absolute organ weight was 19% lower (*p* = 0.027) in GK compared with WT rats (Figure 5A). However, this difference was not maintained after normalization to body weight (Figure 5B). Morphometric analysis revealed no statistically significant differences between GK and WT rats in the cortical and paracortical regions (Figure 5C), medullary region (Figure 5D), lymphoid follicle area (Figure 5E), or germinal center area (Figure 5F).

### 3.4 Thymic Involution in GK Rats Occurs at Baseline and Is Independent of Antigen Exposure

**FIGURE 6:**
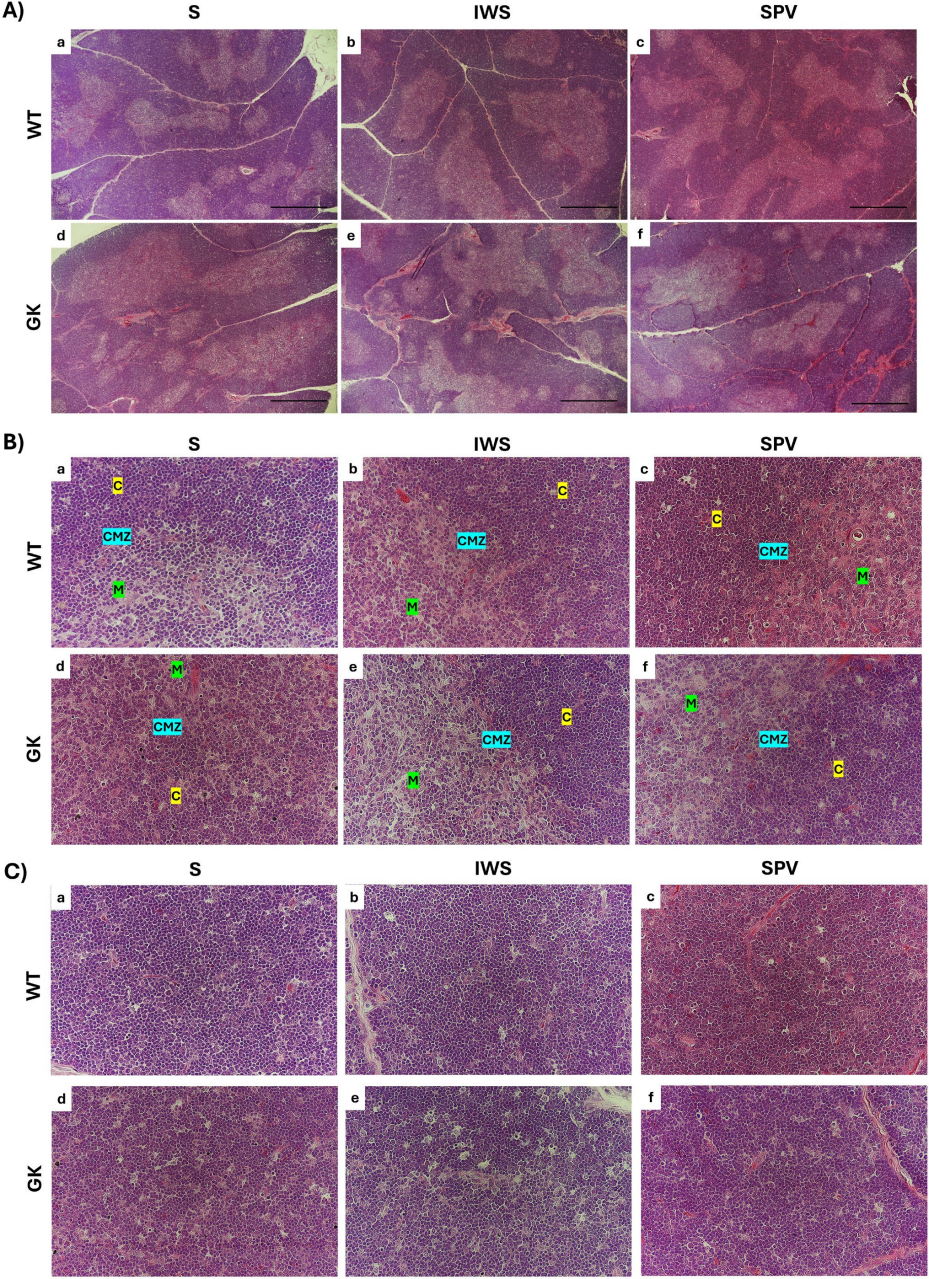
Histopathological evaluation of the thymus in Wistar (WT) and Goto‐Kakizaki (GK) rats following immunization with saline (S), inactivated whole SARS‐CoV‐2 virus (IWS), or spike protein carrying vector (SPV). All analyses were performed in sixteen‐week‐old rats, with thymus collected fifteen days after completion of the immunization protocol. (A) Thymic architecture with preserved lobular organization in WT and GK. Black arrows indicate thickened connective tissue septa in GK rats. (B) Corticomedullary junction zone (CMZ), cortical region (C), and medullary region (M). Note the reduced demarcation of this region in GK rats. (C) Thymic cortex illustrating altered cortical cellularity and the characteristic “starry sky” appearance in GK groups. Scale bars: (A) 500 μm; (B, C) 100 μm.

The thymus of GK rats exhibited a preserved structural organization, characterized by a connective tissue capsule and a lobular architecture with distinct cortical and medullary regions compared with WT (Figure 6). At baseline, GK rats exhibited morphological differences in the thymus relative to WT, including thickening of the connective tissue septa (Figure 6A), a mild loss of demarcation at the corticomedullary junction (Figure 6B), and altered cellularity in the thymic cortex (Figure 6C). The thymic cortex exhibited the characteristic ‘starry sky’ appearance, resulting from an abundance of tangible body macrophages actively clearing apoptotic thymocytes.

**FIGURE 7:**
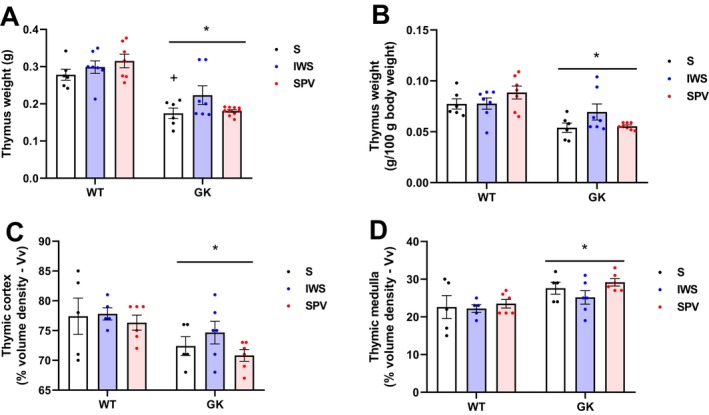
Morphometric analysis of the thymus in Wistar (WT) and Goto‐Kakizaki (GK) rats following immunization with saline (S), inactivated whole SARS‐CoV‐2 virus (IWS), or spike protein carrying vector (SPV). All analyses were performed in 16‐week‐old rats, with thymus collected 15 days after completion of the immunization protocol. (A) Absolute Thymus weight (g). (B) Thymus weight normalized to body mass (g/100 g body weight). (C) Volume density (%Vv) of thymic cortex. (D) Volume density (%Vv) of the thymic medulla. Results are presented as mean ± SEM (*) *p* < 0.05: between strains.

Morphometric analysis revealed significant strain differences in thymic parameters, irrespective of the antigen exposure (Figure 7). Absolute thymus weight was significantly lower in GK compared with WT rats (*p* < 0.0001) (Figure 7A). This difference was maintained after normalization to body weight, corresponding to a 23.6% reduction relative to the thymus weight of WT rats (*p* < 0.0001) (Figure 7B). GK rats exhibited a modest but statistically significant reduction in cortical volume, corresponding to a 5.89% decrease compared with the WT group (*p* = 0.0033) (Figure 7C). In contrast, the medullary compartment was significantly increased in GK, 20.0% higher than that observed in WT rats (*p* = 0.0028) (Figure 7D).

### 3.5 Baseline Splenic Remodeling in GK Rats Is Characterized by Splenomegaly, Red Pulp Expansion, and Compartment‐Specific Alterations in White Pulp Architecture

**FIGURE 8:**
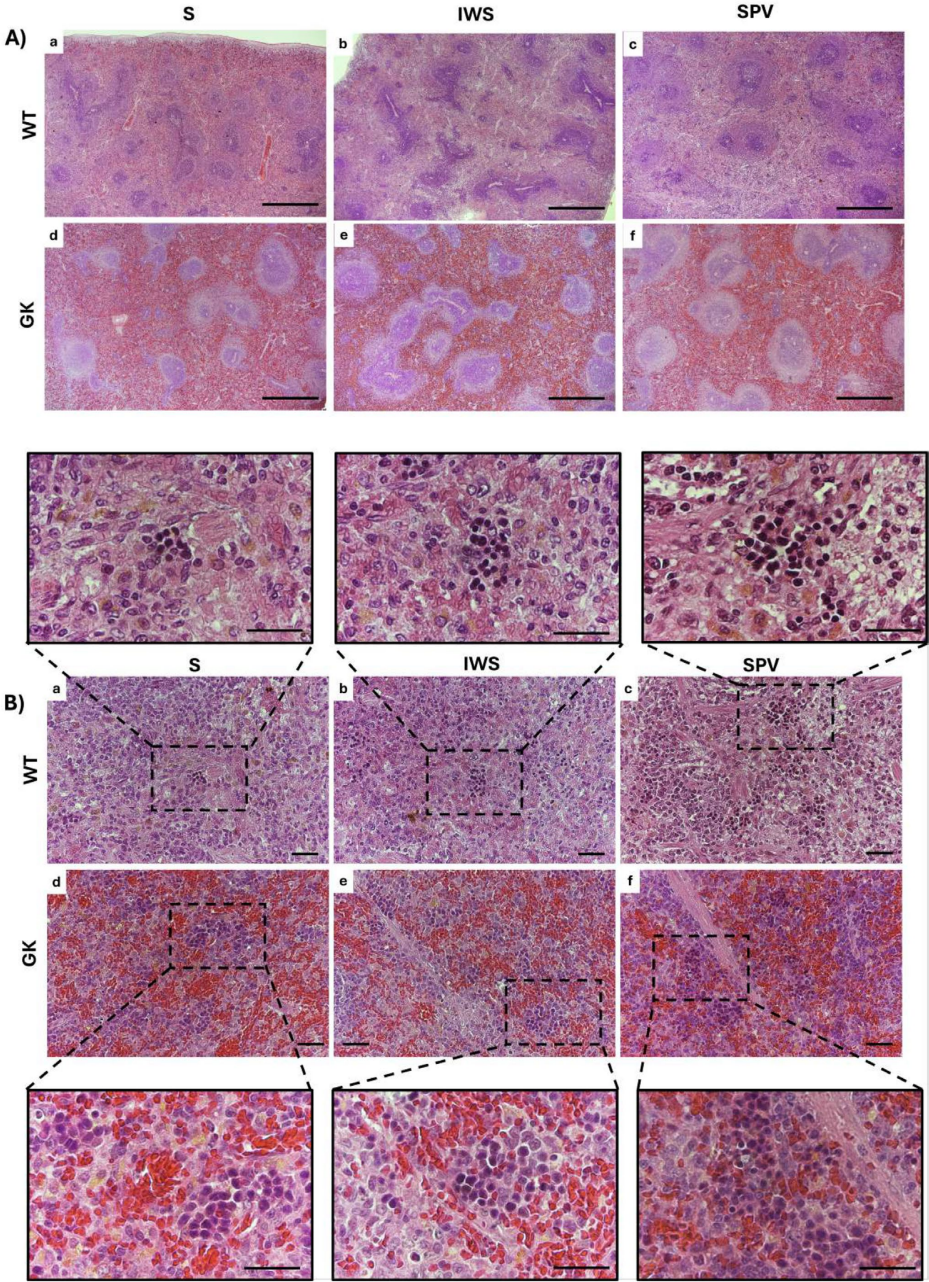
Histopathological evaluation of the spleen in Wistar (WT) and Goto‐Kakizaki (GK) rats following immunization with saline (S), inactivated whole SARS‐CoV‐2 virus (IWS), or spike protein carrying vector (SPV). All analyses were performed in sixteen‐week‐old rats, with spleen collected fifteen days after completion of the immunization protocol. (A) Splenic architecture with defined white and red pulp regions. (B) Red pulp shows preserved structure in WT rats, while GK rats display distended sinusoids and numerous clusters of erythroid precursor cells. High‐magnification details of erythropoietic foci within the red pulp of WT and GK animals across all experimental conditions. Scale bars: (A) 500 μm; (B) 100 μm; high‐magnification 50 μm.

WT and GK rats exhibited preserved splenic architecture, with clearly defined white and red pulp compartments (Figure 8A). However, the relative proportions of these compartments differed between groups, particularly in the distribution of white pulp subregions. In GK rats, the red pulp displayed distended sinusoids filled with erythrocytes and numerous clusters of erythroid precursor cells, consistent with extramedullary hematopoiesis (Figure 8B).

**FIGURE 9:**
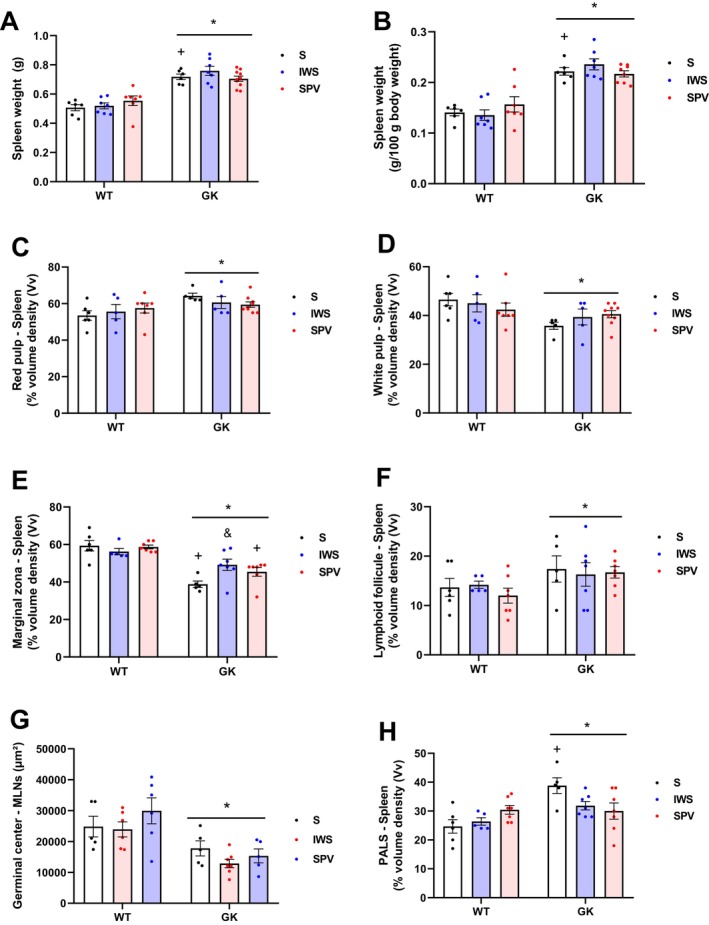
Morphometric analysis of the spleen in Wistar (WT) and Goto‐Kakizaki (GK) rats following immunization with saline (S), inactivated whole SARS‐CoV‐2 virus (IWS), or spike protein carrying vector (SPV). All analyses were performed in sixteen‐week‐old rats, with spleen collected fifteen days after completion of the immunization protocol. (A) Absolute spleen weight (g). (B) Spleen weight normalized to body mass (g/100 g body weight). (C) Volume density (%Vv) of red pulp. (D) Volume density (%Vv) of white pulp. (E) Volume density (%Vv) of marginal zone. (F) Volume density (%Vv) of lymphoid follicle. (G) Volume density (%Vv) of germinal center. (H) Volume density (%Vv) of periarteriolar lymphoid sheath (PALS). Results are presented as mean ± SEM (*) *p* < 0.05: between strains; (#) *p* < 0.05: between immunized animals (same strain); (&) *p* < 0.05: versus saline group (same strain); (+) *p* < 0.05: same treatment, different strains.

Absolute spleen weight was higher in GK than in WT rats (*p* < 0.0001) (Figure 9A). This difference persisted after normalization to body weight, with GK displaying a 55.9% higher relative spleen weight than WT rats (*p* < 0.0001) (Figure 9B). This pattern was not altered during antigen exposure, and all GK groups showed greater splenic mass than their corresponding WT groups (*p* < 0.0001).

Morphometric analysis of splenic tissue compartments revealed strain‐dependent differences. GK rats exhibited a 10.6% higher red pulp volume density compared with WT animals (*p* = 0.0098) (Figure 9C), consistent with the sinusoidal congestion and erythroid precursor clusters observed histologically. This alteration was accompanied by a 13.6% reduction in total white pulp volume density in GK rats relative to WT controls (*p* = 0.0064) (Figure 9D).

In white pulp, marginal zone volume density was reduced by 23.5% (*p* < 0.0001) at baseline in GK compared with WT rats (Figure 9E). This strain difference was maintained following antigen exposure. In post hoc analyses, marginal zone volume was lower in GK‐S animals than in GK‐IWS (*p* = 0.0416) and was also reduced in GK‐S and GK‐SPV compared with the corresponding WT groups (WT‐S, *p* < 0.0001; WT‐SPV, *p* = 0.0016).

Despite the overall reduction in white pulp volume, specific subregions were increased in GK animals. The lymphoid follicle compartment was 26.4% greater in GK rats compared with WT animals (*p* = 0.0267) (Figure 9F), and the germinal center compartment was elevated by 152% compared with WT (*p* = 0.0002) (Figure 9G). GK rats exhibited larger PALS than WT animals. Overall, PALS volume density was 23.5% higher in GK than in WT rats (*p* = 0.0010). In saline‐treated animals, PALS volume density was significantly greater in GK than in WT rats (≈57.3% difference; *p* = 0.0014), and this strain difference was not altered by antigen exposure (Figure 9H).

## 4 Discussion

T2DM is a significant risk factor for COVID‐19 and one of the most prevalent comorbidities among non‐survivors [31, 32]. Furthermore, the diabetic state can impair the immune response to vaccines [33]. Evidence suggests that the effectiveness of COVID‐19 vaccines in individuals with T2DM, considering infection prevention, symptomatic disease, hospitalization, and mortality outcomes, is lower than that reported in non‐diabetic patients [34]. The literature indicates different responses to the combination of T2DM and COVID‐19, as well as impaired post‐vaccination immunity, with considerable variability across studies [10].

Despite advances in understanding the relationship between T2DM and vaccine responses, significant gaps remain concerning the impact of T2DM on vaccine immunogenicity in the absence of obesity. The primary aim of this study was to investigate whether T2DM, independent of obesity, modulates the morphology of primary and secondary lymphoid organs and how these changes influence IgG production following immunization with SARS‐CoV‐2 antigens.

To this end, we used GK rats, a non‐obese experimental model of T2DM [12]. The present study was conducted using the GK rat colony maintained by our research group. The GK breeding stock maintained at the Animal Welfare Center of the Butantan Institute (São Paulo, Brazil) was originally imported from Taconic Bioscience to establish our GK colony and has since been bred locally for nine generations. We are currently undergoing comprehensive phenotype, metabolic, immunological, and genetic characterization of our GK colony.

Our GK colony indicates early‐onset hyperglycemia and insulin resistance, accompanied by metabolic and immune alterations. Recent studies from this colony have demonstrated elevated HOMA‐IR indices as early as 21 days of age, along with early metabolic alterations in skeletal muscle and in amino acid and lipid metabolism [35]. In the present study, the morphometabolic characteristics of GK rats were consistent with those previously described [13, 15]. Compared with normoglycemic WT, GK rats exhibited lower body mass, reduced naso–anal and tibial lengths, smaller abdominal circumference and fat deposits, and pronounced fasting hyperglycemia during the GTT. These findings validate this model for investigating diabetes‐induced immunological alterations independent of obesity.

Hyperglycemia in GK rats constitutes a key component of the pathophysiology of this experimental model. Hyperglycemia creates an adverse metabolic state that promotes oxidative stress and pro‐inflammatory signaling in tissues, thereby compromising immune function [36, 37]. This metabolic condition likely contributed to the observed morphological and morphometric changes, including thymic involution and splenic architectural remodeling. Still, it did not reduce antibody production reported at the time point analyzed.

In the Goto‐Kakizaki (GK) rat model, immune dysfunction can vary with animal age and across biological compartments, underscoring the need for context‐dependent interpretation of immunological findings. Previous studies have shown that systemic immune parameters assessed in adult GK rats may not display signatures of systemic inflammation, including altered circulating immune cell numbers and reduced levels of peripheral cytokines [16]. In parallel, tissue‐ and cell‐specific immune analyses have revealed distinct immunological alterations within lymphoid and peripheral compartments. Macrophages isolated from GK rats exhibit a bias toward a pro‐inflammatory M1 phenotype with reduced M2 polarization [17], and mesenteric lymph node lymphocytes display increased T‐cell activation and impaired differentiation, characterized by a predominance of T helper 1 cells and reduced T helper 2 and regulatory T‐cell populations [18]. Additional studies performed in younger GK rats reported elevated plasma inflammatory markers, such as TNF‐α and C‐reactive protein, associated with immunological alterations in the thymus and spleen, including reduced lymphocyte proliferation, thymic atrophy, increased oxidative stress, mitochondrial dysfunction, and activation of local apoptotic pathways in thymocytes [36].

GK rats exhibited higher anti‐Spike IgG titers than normoglycemic WT controls, regardless of the antigen formulation. Although contrary to our initial hypothesis, this result is consistent with previous reports describing enhanced antibody production in the context of diabetes. Takeda and Wakabayashi [37] demonstrated increased antigen‐specific IgG2a production in young GK rats following ovalbumin immunization. Frasca et al. [38] reported robust humoral responses to influenza vaccination in both young and elderly individuals with T2DM and elevated levels of systemic inflammatory cytokines. Zhai et al. [39] described that T2DM patients exhibit increased immunoglobulin production, accompanied by elevated basal B‐cell activation and enhanced expression of co‐stimulatory molecules, including CD86 and CD95. Together, these studies indicate that the diabetic milieu may enhance antibody production through heightened basal B‐cell activation, supporting the hypothesis that metabolic inflammation reshapes the humoral immune response. However, such increases in antibody levels do not necessarily reflect optimal adaptive immune function. Despite the elevated humoral response observed in 16‐week‐old GK rats 15 days after three antigen doses, further studies evaluating antibody virus‐neutralization capacity, longitudinal antibody persistence, and memory T and B‐cell responses are required to elucidate antibody functionality and the immunogenicity in non‐obese diabetes.

To better understand the enhanced humoral response in GK rats, we investigated whether morphometric alterations in lymphoid organs could account for the increased antibody production. To our knowledge, this is the first study to describe the histological and morphometric features of lymphoid organs in GK rats.

MLNs from GK did not differ from WT rats in organ weight or in the morphometric parameters studied, including the cortical, paracortical, and medullary compartments, as well as lymphoid follicle and germinal center areas, after appropriate normalization for organ size. In 16‐week‐old GK rats from our colony, MLN architecture appeared similar to that of WT, indicating that the enhanced antibody production observed in this model is not associated with structural remodeling of the lymph nodes. A limitation of the present study is that the mesenteric lymph nodes analyzed do not correspond to the primary draining lymph nodes of the immunization site. Therefore, while MLNs provide relevant information regarding systemic immune alterations in GK rats, they may not fully capture vaccine‐induced structural or cellular changes occurring in regional draining lymph nodes. This anatomical consideration may partially explain the absence of detectable morphometric alterations in MLNs despite the enhanced humoral responses observed following immunization.

Our group has previously investigated MLNs, focusing on immune cellularity, and demonstrated altered mesenteric lymph node cellular composition and a proinflammatory T‐lymphocyte profile in GK rats. Lobato et al. [18] reported increased T‐cell activation, skewing toward a Th1 phenotype, reduced regulatory T‐cell markers, and enhanced proliferative capacity of MLN‐derived lymphocytes across T2DM progression in GK rats. These findings suggest that diabetes may affect immune cell composition and activation status in MLNs without necessarily inducing detectable changes in lymph node architecture or compartmental organization.

Thymic histology demonstrated features of premature involution. Although the lobular architecture remained intact, there was septal thickening, corticomedullary attenuation, reduction in cortical cellularity, and significant cortical macrophage infiltration. Morphometric analysis confirmed decreased thymic mass, cortical thinning, and medullary expansion. These thymic findings align with prior studies using streptozotocin‐induced diabetes models, which have described rapid thymic involution accompanied by macrophage hyperplasia, cortical thymocyte apoptosis, and oxidative stress [40]. Yang et al. [41] reported diet‐induced obesity‐associated premature thymic involution, with thymocyte reduction, increased apoptosis, and disrupted thymic cellularity in mice. These findings indicate that metabolic stress associated with chronic hyperglycemia can drive premature thymic involution in GK rats, independent of obesity.

The thymic involution observed in GK rats may have important implications for systemic immune homeostasis. Thymic atrophy is known to impair thymopoiesis and reduce the output of naïve T cells, thereby contributing to decreased peripheral lymphocyte availability [42]. Although circulating immune cells were not directly quantified in the present study, thymic involution in GK rats may be associated with alterations in peripheral immune cell availability previously reported in GK rats [16]. Together, these observations support the notion that thymic involution in non‐obese T2DM may represent a link between metabolic dysregulation and systemic immune imbalance, warranting further investigation.

In the spleen, although GK rats exhibited an overall reduction in splenic white pulp, regional analysis revealed a compartment‐specific remodeling. The marginal zone, which plays a pivotal role in rapid B‐cell‐mediated responses against bacterial antigens [43], was contracted, suggesting a potential vulnerability in innate‐like antibacterial defense. In contrast, the lymphoid follicle, germinal center, and PALS were expanded. Germinal centers are sites of antigen‐driven B‐cell activation, proliferation, and differentiation [44], whereas the PALS is enriched in T cells [20]. This shift in splenic architecture may reflect an adaptive reorganization in which the loss of marginal zone integrity promotes greater reliance on germinal center pathways, thereby sustaining enhanced antibody generation [45]. Such remodeling provides a structural context that may help explain the elevated anti‐Spike IgG titers observed in GK rats. Atef et al. [46] demonstrated that diabetic mouse models (ob/ob and STZ‐treated) displayed splenic histological remodeling characterized by reduced white pulp and T‐ and B‐cell zones, which was associated with impaired antibody persistence [46] after SARS‐CoV‐2 vaccination. These findings highlight that, while diabetes drives splenic remodeling, the specific patterns may differ between obese and non‐obese models, with distinct consequences for the quality and durability of humoral responses. We also observed enhanced extramedullary hematopoiesis in GK rats. While physiological in rodents, its amplification in GK rats may be linked to T2DM, since extramedullary hematopoiesis is typically activated to compensate for bone marrow deficiencies during inflammatory stress.

In summary, we reported that non‐obese T2DM GK rats exhibit alterations in the morphology of both primary and secondary lymphoid organs, leading to early thymic involution and splenic remodeling. These findings indicate that GK rats exhibit a notable remodeling of the systemic immune organization. The structural alterations reported in lymphoid organs did not suppress antibody production during the period investigated; rather, GK rats exhibited higher anti‐Spike IgG titers than normoglycemic controls. This apparent dissociation between lymphoid architecture and antibody titers in GK rats needs further studies to assess IgG functionality and the activation dynamics of adaptive immune cells.

## 5 Conclusion

Despite marked morphological differences in lymphoid organs, GK rats displayed an enhanced humoral response, evidenced by elevated anti‐Spike IgG titers under antigen exposure. The present study supports investigations into the antigen‐neutralizing capacity of antibodies, T‐ and B‐cell memory responses, and the persistence of immunization protection following vaccination in non‐obese T2DM.

## Author Contributions

Conceptualization and design of the work: M.C.L, M.L.S., S.C.S.V., V.G.M.M, and R.C.; Acquisition of data: S.A.P, and M.C.L.; Analysis and interpretation of data: S.A.P, M.C.L, B.C.L.F.T, E.F.L.M, M.L.Z.D., and R.C.; Writing of the original draft: S.A.P, M.C.L, and R.C.; Revising critically the work: M.L.Z.D, L.R.C.G, B.C.L.F.T, E.F.L.M, V.G.M.M, and R.C.; Acquisition of resources: M.S.S, R.N.O, S.C.S.V., and R.C.; Supervision: R.C. All authors were involved in drafting and revising the manuscript.

## Funding

This work was supported by the São Paulo State Research Support Foundation (FAPESP: 2024/08439‐6); the National Research and Development Council (CNPq: 403695/2023‐6); CeVIVAS Project funded by the São Paulo Research Foundation (FAPESP: 2021/11944‐6); and Fundação Butantan.

## Conflicts of Interest

The authors declare no conflicts of interest.

## Supporting information


**Table S1:** Glucose tolerance test (GTT, mg/dL) was performed with glucose measurements at 0 min (before injection) and at 15, 30, and 60 min after intraperitoneal administration of 50% glucose solution (2 g/kg) in WT‐S, WT‐IWS, WT‐SPV, GK‐S, GK‐IWS, and GK‐SPV. Wistar (WT) and Goto‐Kakizaki (GK) rats, following immunization with saline (S), inactivated whole SARS‐CoV‐2 virus (IWS), or spike protein carrying vector (SPV).
**Table S2:** Serum IgG levels against the spike protein were evaluated by ELISA using serial dilutions in Wistar (WT) and Goto‐Kakizaki (GK) rats following immunization with saline (S) inactivated whole SARS‐CoV‐2 virus (IWS) or spike protein carrying vector (SPV).
**Table S3:** Morphometric analysis of the mesenteric lymph nodes (MLNs) in Wistar (WT) and Goto‐Kakizaki (GK) rats following immunization with saline (S), inactivated whole SARS‐CoV‐2 virus (IWS), or spike protein carrying vector (SPV). Body mass (g); Absolute MLNs mass (MLN/g); MLN mass normalized to 100 g of body mass (g/100 g); Volume density (%Vv) of cortical and paracortical regions; Volume density (%Vv) of medullary region; Total area of lymphoid follicles (μm^2^); Lymphoid follicle area normalized to MLN mass (mm^2^/g); Total area germinal center (μm^2^); Germinal center area normalized to MLN mass (mm^2^/g).
**Table S4:** Morphometric analysis of the thymus in Wistar (WT) and Goto‐Kakizaki (GK) rats following immunization with saline (S), inactivated whole SARS CoV‐2 virus (IWS), or spike protein carrying vector (SPV). Body mass (g); Absolute thymus mass (g); Thymus mass normalized to 100 g of body mass (g/100 g); Volume density (%Vv) of thymic cortex; and Volume density (%Vv) of the thymic medulla.
**Table S5:** Morphometric analysis of the spleen in Wistar (WT) and Goto‐Kakizaki (GK) rats following immunization with saline (S), inactivated whole SARS CoV‐2 virus (IWS), or spike protein carrying vector (SPV). Relative spleen weight normalized by tibia length; Volume density (%Vv) of red pulp; Volume density (%Vv) of white pulp; Volume density (%Vv) of marginal zone; Volume density (%Vv) of lymphoid follicle; Volume density (%Vv) of germinal center; Volume density (%Vv) of periarteriolar lymphoid sheath (PALS).

## Data Availability

The data that support the findings of this study are available in the Supporting Information of this article.
