# Supplementary material for: Lymphoid Organs Remodeling in Non‐Obese Diabetic Goto‐Kakizaki Rats Immunized With SARS‐CoV‐2 Antigens
Source: FASEB J. 2026 Jan 24;40(2):e71493. doi: 10.1096/fj.202503631R (PMC12831124; doi:10.1096/fj.202503631R)
Supplement: Supplementary file 1 — Table S1: Glucose tolerance test (GTT, mg/dL) was performed with glucose measurements at 0 min (before injection) and at 15, 30, and 60 min after intraperitoneal administration of 50% glucose solution (2 g/kg) in WT‐S, WT‐IWS, WT‐SPV, GK‐S, GK‐IWS, and GK‐SPV. Wistar (WT) and Goto‐Kakizaki (GK) rats, following immunization with saline (S), inactivated whole SARS‐CoV‐2 virus (IWS), or spike protein carrying vector (SPV). Table S2: Serum IgG levels against the spike protein were evaluated by ELISA using serial dilutions in Wistar (WT) and Goto‐Kakizaki (GK) rats following immunization with saline (S) inactivated whole SARS‐CoV‐2 virus (IWS) or spike protein carrying vector (SPV). Table S3: Morphometric analysis of the mesenteric lymph nodes (MLNs) in Wistar (WT) and Goto‐Kakizaki (GK) rats following immunization with saline (S), inactivated whole SARS‐CoV‐2 virus (IWS), or spike protein carrying vector (SPV). Body mass (g); Absolute MLNs mass (MLN/g); MLN mass normalized to 100 g of body mass (g/100 g); Volume density (%Vv) of cortical and paracortical regions; Volume density (%Vv) of medullary region; Total area of lymphoid follicles (μm2); Lymphoid follicle area normalized to MLN mass (mm2/g); Total area germinal center (μm2); Germinal center area normalized to MLN mass (mm2/g). Table S4: Morphometric analysis of the thymus in Wistar (WT) and Goto‐Kakizaki (GK) rats following immunization with saline (S), inactivated whole SARS CoV‐2 virus (IWS), or spike protein carrying vector (SPV). Body mass (g); Absolute thymus mass (g); Thymus mass normalized to 100 g of body mass (g/100 g); Volume density (%Vv) of thymic cortex; and Volume density (%Vv) of the thymic medulla. Table S5: Morphometric analysis of the spleen in Wistar (WT) and Goto‐Kakizaki (GK) rats following immunization with saline (S), inactivated whole SARS CoV‐2 virus (IWS), or spike protein carrying vector (SPV). Relative spleen weight normalized by tibia length; Volume density (%Vv) of red pulp; Volume de [file FSB2-40-e71493-s001.pdf]

SUPPLEMENTAL DATA

Table S1. Glucose tolerance test (GTT, mg/dL) was performed with glucose measurements at 0 min (before injection) and at 15, 30, and 60 min after intraperitoneal administration of 50% glucose solution (2 g/kg) in WT-S, WT-IWS, WT-SPV, GK-S, GK-IWS, and GK-SPV. Wistar (WT) and Goto-Kakizaki (GK) rats following immunization with saline (S), inactivated whole SARS-CoV-2 virus (IWS), or spike protein carrying vector (SPV).

**Glucose Tolerance Test (GTT)**											
**Animal number**		1	2	3	4	5	6	7	8	9	10
**Group**	**Time (m)**	**Glucose concentration (mg/dL)**									
**WT-S**	**0**	106	120	97	105	126	119	-	-	-	-
	**15**	172	260	220	218	350	212	-	-	-	-
	**30**	155	194	219	197	299	188	-	-	-	-
	**60**	130	128	134	144	223	144	-	-	-	-
	**90**	110	126	93	120	186	135	-	-	-	-
**WT-IWS**	**0**	112	89	122	108	122	104	121	-	-	-
	**15**	304	232	225	215	249	232	223	-	-	-
	**30**	258	169	208	192	212	214	205	-	-	-
	**60**	146	125	148	124	172	136	167	-	-	-
	**90**	121	106	126	121	149	111	153	-	-	-
**WT-SPV**	**0**	118	113	107	100	115	126	102	-	-	-
	**15**	244	232	242	182	202	219	174	-	-	-
	**30**	165	187	203	196	185	223	173	-	-	-

	**60**	138	132	125	113	155	195	155	-	-	-
	**90**	115	107	123	109	137	167	167	-	-	-
**GK-S**	**0**	132	159	190	204	198	188	-	-	-	-
	**15**	404	498	509	430	565	519	-	-	-	-
	**30**	488	482	505	477	411	543	-	-	-	-
	**60**	397	449	418	439	368	433	-	-	-	-
	**90**	373	415	416	388	313	389	-	-	-	-
**GK-IWS**	**0**	141	147	138	154	216	163	178	-	-	-
	**15**	516	459	494	295	508	406	533	-	-	-
	**30**	484	491	495	291	539	425	513	-	-	-
	**60**	414	410	403	297	463	397	425	-	-	-
	**90**	311	420	414	312	411	399	398	-	-	-
**GK-SPV**	**0**	225	179	168	191	224	174	171	161	207	224
	**15**	472	503	503	490	416	431	332	442	413	535
	**30**	539	511	508	512	437	476	389	423	494	556
	**60**	486	451	482	450	358	406	408	375	505	433
	**90**	434	412	467	426	288	414	422	396	436	389

Table S2. Serum IgG levels against the spike protein were evaluated by ELISA using serial dilutions in Wistar (WT) and Goto-Kakizaki (GK) rats following immunization with saline (S), inactivated whole SARS-CoV-2 virus (IWS), or spike protein carrying vector (SPV).

**ELISA IgG anti-spike**							
**Animal number**		1	2	3	4	5	6
**Group**	**Dilution**	**Optic Density (492nm)**					
**WT-S**	**200**	0,00835	0,01145	0,03000	0,03000	0,02325	0,01425
	**400**	0,00795	0,00595	0,01000	0,02000	0,00815	0,00615
	**800**	0,00775	0,00755	0,01000	0,01000	0,00745	0,00475
	**1600**	0,00455	0,00355	0,00000	0,01000	0,00315	0,00365
	**3200**	0,00425	0,02415	-0,02000	-0,02000	0,00095	0,00125
	**6400**	0,00005	0,00085	0,00000	0,00000	0,00065	0,00075
	**12800**	0,00085	0,00185	0,00000	0,00000	0,00065	0,00185
	**25600**	0,00325	0,00485	0,00000	0,01000	0,00205	0,00295
**WT-IWS**	**200**	1,05445	0,77425	1,06000	1,05000	1,52615	1,83355
	**400**	1,19295	0,86615	0,99000	1,01000	1,36805	1,95355
	**800**	1,01975	0,65315	0,80000	0,66000	1,19065	1,81955
	**1600**	0,86445	0,36625	0,43000	0,40000	0,90765	1,54325
	**3200**	0,61555	0,25095	0,28000	0,23000	0,63955	1,17565
	**6400**	0,39505	0,14135	0,16000	0,15000	0,42395	0,81915
	**12800**	0,22995	0,06595	0,09000	0,08000	0,23085	0,50955
	**25600**	0,12295	0,03715	0,05000	0,04000	0,11685	0,25885

**WT-SPV**	**200**	1,53775	1,49055	1,87000	1,65000	2,02485	1,91495
	**400**	1,66095	1,36135	2,00000	1,81000	1,63735	1,82095
	**800**	1,47485	1,21525	1,75000	1,73000	1,28165	1,80045
	**1600**	0,98925	0,83405	1,29000	1,26000	0,81075	1,22705
	**3200**	0,75485	0,62305	0,99000	0,83000	0,56335	1,03205
	**6400**	0,49475	0,40045	0,75000	0,58000	0,37885	0,72425
	**12800**	0,28035	0,20765	0,50000	0,33000	0,22595	0,45055
	**25600**	0,14125	0,10635	0,26000	0,15000	0,09205	0,23725
**GK-S**	**200**	0,01885	0,01475	0,01000	0,04000	0,02875	-
	**400**	0,00855	0,00975	0,01000	0,02000	0,01385	-
	**800**	0,00695	0,00585	0,01000	0,01000	0,00825	-
	**1600**	0,00525	0,00395	0,00000	0,01000	0,00455	-
	**3200**	0,00095	-0,00095	-0,02000	-0,02000	0,00205	-
	**6400**	0,00175	-0,00105	0,00000	0,00000	0,00385	-
	**12800**	0,00265	0,00015	0,00000	0,00000	0,00415	-
	**25600**	0,00085	-0,00115	0,00000	0,00000	0,00175	-
**GK-IWS**	**200**	1,59435	1,58255	2,12000	1,64000	1,83325	-
	**400**	1,64375	1,59605	1,62000	1,92000	1,69615	-
	**800**	1,61745	1,47565	2,11000	1,85000	1,84435	-
	**1600**	1,36545	1,22215	1,60000	1,20000	1,30505	-
	**3200**	1,21085	0,96675	1,51000	1,02000	1,33555	-
	**6400**	1,06745	0,62055	1,13000	0,90000	0,97455	-
	**12800**	0,65335	0,36365	0,69000	0,51000	0,59395	-
	**25600**	0,37095	0,20655	0,43000	0,22000	0,30335	-
**GK-SPV**	**200**	1,83465	2,27000	1,92075	1,83605	2,10785	-
	**400**	1,68805	1,90000	1,90615	1,88805	1,71195	-

	**800**	1,63645	1,83000	1,80225	1,87125	1,69645	-
	**1600**	1,42835	1,55000	1,75635	1,52415	1,34185	-
	**3200**	1,25195	1,22000	1,49425	1,32365	1,19785	-
	**6400**	0,87305	0,86000	1,04875	0,88985	0,74825	-
	**12800**	0,53825	0,61000	0,72845	0,58985	0,55005	-
	**25600**	0,34735	0,38000	0,43235	0,37925	0,26375	-

Table S3. Morphometric analysis of the mesenteric lymph nodes (MLNs) in Wistar (WT) and Goto-Kakizaki (GK) rats following immunization with saline (S), inactivated whole SARS-CoV-2 virus (IWS), or spike protein carrying vector (SPV). Body mass (g); Absolute MLNs mass (MLN/g); MLN mass normalized to 100 g of body mass(g/100g); Volume density (%Vv) of cortical and paracortical regions; Volume density (%Vv) of medullary region; Total area of lymphoid follicles (μm^2); Lymphoid follicle area normalized to MLN mass (mm^2/g); Total area germinal center (μm^2); Germinal center area normalized to MLN mass (mm^2/g).

Morphometric analysis of mesenteric lymph nodes										
**Group**	**Animal**	**Body mass (g)**	**MLN (g)**	**MLN (g/100g body mass)**	**% Vv cortex+paracortex**	**% Vv medula**	**Σ Lymphoid follicule (μm²)**	**Lymphoid follicule (mm²/g)**	**Σ Germinal center (μm²)**	**Germinal center (mm²/g)**
**WT-S**	**1**	320	0,368	0,115	52	48	-	-	-	-
	**2**	349	0,365	0,105	71	29	1103360	3,023	293374	0,804
	**3**	356	0,4	0,112	66	34	1333625	3,334	255995	0,640
	**4**	348	0,4	0,115	81	19	1789148	4,473	432810	1,082
	**5**	413	0,237	0,057	81	19	3846614	16,230	452480	1,909
	**6**	386	0,343	0,089	-	-	847092	2,470	147473	0,430
**WT-IWS**	**1**	392	0,363	0,093	77	22	2160028	5,950	302659	0,834
	**2**	334	0,455	0,136	71	27	1864993	4,099	205776	0,452
	**3**	338	0,42	0,124	80	19	1383287	3,294	397248	0,946
	**4**	366	0,169	0,046	65	35	-	-	-	0,000
	**5**	434	0,211	0,049	83	17	958803	4,544	119624	0,567
	**6**	422	0,291	0,069	66	34	2941732	10,109	354178	1,217
	**7**	445	0,511	0,115	-	-	2255267	4,413	438536	0,858
**WT-SPV**	**1**	374	0,411	0,110	74	26	2910803	7,082	989464	2,407
	**2**	302	0,569	0,188	77	23	1603378	2,818	127331	0,224

	**3**	291	0,428	0,147	66	34	2445652	5,714	599701	1,401
	**4**	360	0,27	0,075	76	24	-	-	-	0,000
	**5**	398	0,342	0,086	73	27	2287731	6,689	396211	1,159
	**6**	385	0,426	0,111	85	15	2532266	5,944	286611	0,673
	**7**	423	0,267	0,063	-	-	2075211	7,772	535576	2,006
**GK-S**	**1**	339	0,336	0,099	88	11	2590421	7,710	447594	1,332
	**2**	335	0,294	0,088	75	25	2072005	7,048	158209	0,538
	**3**	300	0,499	0,166	76	24	1770221	3,548	325191	0,652
	**4**	352	0,333	0,095	76	24	-	-	-	0,000
	**5**	309	0,157	0,051	88	12	2389531	15,220	290591	1,851
	**6**	312	0,205	0,066	-	-	1827411	8,914	252686	1,233
**GK-IWS**	**1**	341	0,348	0,102	82	18	1521500	4,372	118023	0,339
	**2**	324	0,398	0,123	78	22	1402019	3,523	62838	0,158
	**3**	327	0,266	0,081	84	17	1466624	5,514	55113	0,207
	**4**	326	0,271	0,083	68	32	1480323	5,462	118849	0,439
	**5**	318	0,235	0,074	69	31	1829738	7,786	237108	1,009
	**6**	313	0,255	0,081	73	27	1720742	6,748	172866	0,678
**GK-SPV**	**1**	308	0,27	0,088	76	24	1551740	5,747	201154	0,745
	**2**	326	0,383	0,117	83	17	1080560	2,821	65710	0,172
	**3**	328	0,328	0,100	73	27	1566629	4,776	252181	0,769
	**4**	306	0,244	0,080	86	14	1888468	7,740	413387	1,694
	**5**	332	0,214	0,064	70	30	1277925	5,972	324491	1,516
	**6**	350	0,299	0,085	-	-	-	-	-	-
	**7**	312	0,246	0,079	-	-	-	-	-	-
	**8**	324	0,263	0,081	-	-	-	-	-	-

Table S4. Morphometric analysis of the thymus in Wistar (WT) and Goto-Kakizaki (GK) rats following immunization with saline (S), inactivated whole SARS-CoV-2 virus (IWS), or spike protein carrying vector (SPV). Body mass (g); Absolute thymus mass (g); Thymus mass normalized to 100 g of body mass(g/100g); Volume density (%Vv) of thymic cortex; and Volume density (%Vv) of the thymic medulla.

**Morphometric analysis of thymus**						
**Group**	**Animal**	**Body mass (g)**	**Thymus (g)**	**Thymus (g/100g body mass)**	**% Vv thymic cortex**	**% Vv Thymic medulla**
**WT-S**	1	320	0,275	0,086	78	22
	2	349	0,242	0,069	85	15
	3	356	0,249	0,070	83	17
	4	348	0,342	0,098	70	30
	5	413	0,271	0,066	71	29
	6	386	0,29	0,075	-	-
**WT-IWS**	1	392	0,341	0,087	77	23
	2	334	0,297	0,089	79	21
	3	338	0,3	0,089	77	23
	4	366	0,299	0,082	75	25
	5	434	0,213	0,049	81	19
	6	422	0,291	0,069	-	-
	7	445	0,35	0,079	-	-
**WT-SPV**	1	374	0,34	0,091	79	21
	2	302	0,257	0,085	79	21
	3	291	0,316	0,109	74	26
	4	360	0,377	0,105	79	21
	5	398	0,273	0,069	72	27
	6	385	0,369	0,096	75	25

	7	423	0,275	0,065	-	-
**GK-S**	1	339	0,198	0,058	71	29
	2	335	0,203	0,061	68	32
	3	300	0,21	0,070	76	24
	4	352	0,148	0,042	76	24
	5	309	0,127	0,041	71	29
	6	312	0,162	0,052	-	-
**GK-IWS**	1	307	0,319	0,104	81	19
	2	341	0,319	0,094	78	22
	3	324	0,207	0,064	75	25
	4	327	0,2	0,061	75	25
	5	326	0,173	0,053	71	29
	6	318	0,174	0,055	68	31
	7	313	0,171	0,055	-	-
**GK-SPV**	1	308	0,183	0,059	73	27
	2	326	0,191	0,059	72	28
	3	328	0,181	0,055	71	29
	4	306	0,168	0,055	73	27
	5	332	0,174	0,052	67	33
	6	350	0,189	0,054	69	31
	7	312	0,158	0,051	-	-
	8	324	0,19	0,059	-	-
	9	338	0,182	0,054	-	-
	10	318	0,192	0,060	-	-

Table S5. Morphometric analysis of the spleen in Wistar (WT) and Goto-Kakizaki (GK) rats following immunization with saline (S), inactivated whole SARS-CoV-2 virus (IWS), or spike protein carrying vector (SPV). Relative spleen weight normalized by tibia length; Volume density (%Vv) of red pulp; Volume density (%Vv) of white pulp; Volume density (%Vv) of marginal zone; Volume density (%Vv) of lymphoid follicle; Volume density (%Vv) of germinal center; Volume density (%Vv) of periarteriolar lymphoid sheath (PALS).

Morphometric analysis of spleen										
**Group**	**Animal**	**Body mass (g)**	**Spleen (g)**	**Spleen (g/100g body mass)**	**% Vv Red Pulp**	**% Vv White Pulp**	**% Vv Marginal zona**	**% Vv Lymphoid foliicule**	**% Vv Germinal center**	**% Vv PALS**
**WT-S**	1	320	0,429	0,134	55	44	57	19	2,53	20
	2	349	0,525	0,150	63	38	60	12	3,68	26
	3	356	0,529	0,149	51	49	69	13	0,84	17
	4	348	0,541	0,155	57	43	64	8	0	28
	5	413	0,457	0,111	44	56	57	19	0	24
	6	386	0,559	0,145	51	49	49	11	0	33
**WT-IWS**	1	392	0,46	0,117	55	45	54	16	1,09	25
	2	334	0,59	0,177	63	37	55	13	2,38	24
	3	338	0,582	0,172	65	38	54	13	2,47	30
	4	366	0,477	0,130	51	49	55	16	1,59	29
	5	434	0,538	0,124	44	56	63	13	0	24
	6	422	0,466	0,110	-	-	-	-	-	-
	7	445	0,523	0,118	-	-	-	-	-	-
**WT-SPV**	1	374	0,578	0,155	60	40	57	18	3,68	36
	2	302	0,58	0,192	60	40	60	7	1,96	31
	3	291	0,659	0,226	60	40	58	9	0,71	31
	4	360	0,377	0,105	66	34	56	16	1,3	35

	5	398	0,567	0,142	55	45	62	12	0	26
	6	385	0,536	0,139	59	41	56	13	0,85	28
	7	423	0,582	0,138	43	57	62	9	1,59	26
**GK-S**	1	339	0,738	0,218	62	38	45	15	2,98	30
	2	335	0,666	0,199	62	38	35	24	2,26	38
	3	300	0,758	0,253	63	37	38	17	5,19	40
	4	352	0,775	0,220	70	30	37	22	4,76	39
	5	309	0,707	0,229	64	36	39	9	2,03	47
	6	312	0,662	0,212	-	-	-	-	-	-
**GK-IWS**	1	307	0,874	0,285	72	28	49	9	4,23	34
	2	341	0,73	0,214	55	45	57	20	4,23	31
	3	324	0,843	0,260	55	45	34	19	0	38
	4	327	0,795	0,243	57	43	48	26	0	28
	5	326	0,75	0,230	64	36	47	18	4,96	28
	6	318	0,673	0,212	-	-	51	9	3,85	35
	7	313	0,647	0,207	-	-	58	13	3,6	29
**GK-SPV**	1	308	0,664	0,216	61	39	48	17	3,36	24
	2	326	0,738	0,226	55	45	49	14	3,48	34
	3	328	0,771	0,235	57	43	48	21	6,38	30
	4	306	0,708	0,231	58	42	32	16	4	38
	5	332	0,784	0,236	57	43	44	12	3,15	38
	6	350	0,7	0,200	63	37	49	18	3,4	18
	7	312	0,62	0,199	69	31	48	19	3,95	28
	8	324	0,625	0,193	60	40	-	-	-	-
	9	338	0,75	0,222	55	45	-	-	-	-
	10	318	0,69	0,217	-	-	-	-	-	-
